# Several Alkaloids in Chinese Herbal Medicine Exert Protection in Acute Kidney Injury: Focus on Mechanism and Target Analysis

**DOI:** 10.1155/2022/2427802

**Published:** 2022-05-13

**Authors:** Yixin Rui, Sheng Li, Fei Luan, Dan Li, Rong Liu, Nan Zeng

**Affiliations:** ^1^State Key Laboratory of Southwestern Chinese Medicine Resources, Chengdu University of Traditional Chinese Medicine, Chengdu, Sichuan 611137, China; ^2^Department of Pharmacology, College of Pharmacy, Chengdu University of Traditional Chinese Medicine, Chengdu, Sichuan 611137, China; ^3^College of Traditional Chinese Materia Medica, Shenyang Pharmaceutical University, Shenyang, Liaoning 110016, China

## Abstract

**Objectives:**

Acute kidney injury (AKI) is a loose set of kidney diseases accompanied by a variety of syndromes, which is a serious threat to human life and health. Some alkaloids are derived from various Chinese herbs have been widely concerned in the improvement of AKI. This review provides the research progress of alkaloids in AKI experimental models and discusses the related molecular mechanisms. *Key Findings*. Alkaloids can protect AKI through various mechanisms including antioxidant stress, improvement of mitochondrial damage, reduction of cell death, induction of autophagy, and inhibition of inflammation. These mechanisms are mainly related to the activation of Nrf2/HO-1 signaling pathway, inhibition of ferroptosis and apoptosis, regulation of PINK1/Parkin pathway, inhibition of TLR4/NF-*κ*B pathway and NLRP3 inflammatory bodies, upregulation of Klotho protein level and so on. In addition, there are a few alkaloids that have certain toxicity on the kidney.

**Conclusion:**

Alkaloids have been shown to significantly improve AKI, but only in pharmacological studies. This paper summarizes the main experimental models currently used in AKI research and describes some representative alkaloids based on recent research. Their potential roles in the prevention and treatment of AKI through different mechanisms are highlighted.

## 1. Introduction

Acute kidney injury (AKI) is a global disease with high morbidity and mortality, which is a potential threat to human life and health. Especially in specific medical units, such as Intensive Care Unit (ICU), cardiac surgery, oncology, and transplant centers, the incidence of AKI may reach 50% or higher [1, 2]. At present, in addition to dialysis, there is no better treatment intervention that can reliably improve survival, inhibit injury or accelerate rehabilitation [3]. There are many factors leading to AKI, including surgery [4, 5], sepsis [6] and most drug-induced renal injuries in clinical application. In short, due to the complex inducement of multiple factors of AKI and different patient groups, many studies have tried to select drugs such as atrial natriuretic peptide [7], fenoldopam [8] and dopamine [9] to prevent the occurrence of AKI. Although some results have been achieved in animal models, there is no definite clinical effect. The treatment of AKI has become a global public problem. The discovery of potential active components for the treatment of AKI is of positive significance to solve this problem [10].

**Figure 1:**
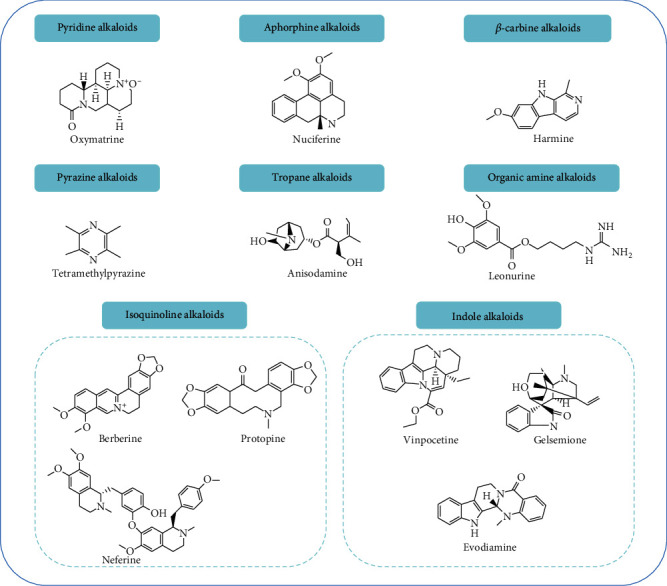
Structural formulas for several alkaloids that exert protection in AKI.

Alkaloids, one of the important effective ingredients in Chinese herbal medicine, are a class of nitrogen-containing organic compounds that exist in nature. Most of alkaloids show obvious anti-inflammatory [11], anti-cancer [12], anti-angiogenic [13] and other evident biological activities, which are widely used in clinical practice. Interestingly, there are many star molecular alkaloids, such as berberine (BBR), that play an extremely important role in kidney diseases [14, 15]. For example, BBR can be used as a potential therapeutic agent for the treatment of diabetic nephropathy in clinical practice [16]. In addition, it plays an distinct role in experimental renal ischemia-reperfusion (I/R) injury [17]. Due to the numerous structural classification of alkaloids, it is difficult to summarize the structural characteristics of all alkaloids. According to the ring structure of alkaloids, the alkaloids that have been found to have the potential to treat AKI are divided into isoquinoline alkaloids, indole alkaloids, pyridine alkaloids, tropane alkaloids, organic amine alkaloids, *β*-carbine alkaloids, aporphine alkaloids, pyrazine alkaloids and so on. The detailed structural formula can be found in Figure 1.

In the current review, we mainly introduce the the clinical etiology of AKI and and illustrate the corresponding experimental model based on these etiologies. Finally, we summarized the protective effects of various alkaloids from Chinese herbal medicines on AKI. This is a review of the protective effects of natural alkaloids and the molecular events involved in different mechanisms to understand their potential improvement of AKI, with a view to providing a theoretical reference for the development of new drugs for the treatment of AKI.

## 2. Clinical Etiology and Experimental Models of AKI

### 2.1. Clinical Etiology of AKI

AKI is not a single disease, but a complex syndrome with multiple underlying causes [18]. Including acute tubular necrosis (ATN) [19], drug-induced acute interstitial glomerulonephritis (AIN) [20], prerenal azotemia [21], hepatorenal syndrome [22] and so on. Clinically, the traditional “anatomical flow” model divides AKI into three types: pre-renal AKI, intrarenal AKI and postrenal AKI [22]. Under the traditional established pattern classification, physicians are often used in the treatment of clinical patients according to this condition [23–25]. However, this traditional classification model based on serum creatinine has inherent defects [26]. In many other clinical AKI cases, there are multiple extrarenal and extrarenal processes, which do not neatly conform to the traditional etiological classification [27]. Therefore, the classification of clinical etiology of AKI needs to be further discussed, and it is more important to determine the etiology of AKI based on clinical evaluation and diagnosis, to provide new ideas for the treatment of AKI.

### 2.2. The Classification of Classical AKI Experimental Models

**Figure 2:**
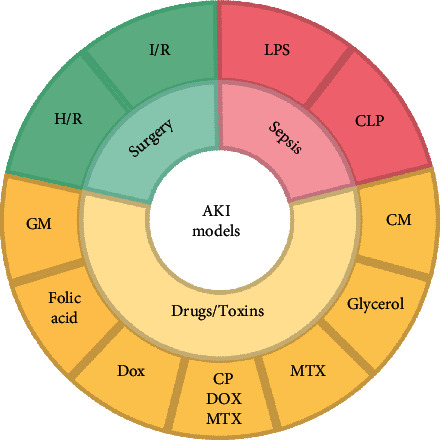
The classification of classical AKI experimental models. These classical models are classified into three categories, according to clinical causes of AKI: surgery, sepsis, or exposure to nephrotoxic substances.(AKI, acute kidney injury; I/R, ischemia/reperfusion; H/R, hypoxia-oxygen-rich; LPS, lipopolysaccharide; CLP, cecal ligation puncture; GM, gentamicin; CP, cisplatin; DOX, doxorubicin; MTX, methotrexate; As, arsenic; RM, rhabdomyolysis; CM, contrast media).

Animal models play an important role in the process of human understanding of diseases [28]. In clinical practice, ATN caused by surgery, sepsis, or exposure to nephrotoxic substances is a widespread cause of AKI [29]. Therefore, most of the current AKI models used in pharmacological experiments are based on this. As we all know, as an indispensable tool to study the pathogenesis of disease, all kinds of experimental models play an essential role in the pathogenesis and pathological process of the disease [26]. A correct understanding of the animal model of disease is helpful for us to better understand the process of disease development [26]. In this part, we summarize three classical models of AKI (Figure 2).

#### 2.2.1. Surgery-AKI Models

As an injury model of kidney, heart, brain, and other diseases, I/R has been widely used in experimental studies [30]. The I/R model mimics AKI caused by operations that sometimes require a temporary interruption of renal blood flow [27]. Unilateral [31] and bilateral [32] I/R-AKI models are the most commonly used animal models at present [33]. Serious structural changes in the kidneys are often found in such models. Similarly, hypoxia-oxygen-rich (H/R) cell models are often used in vitro [34].

#### 2.2.2. Sepsis-AKI Models

Sepsis is a systemic inflammatory response syndrome caused by the invasion of bacteria and other pathogenic microorganisms into the body [35]. It further shows multiple organ dysfunction, including renal organ dysfunction [36]. More seriously, this will lead to an increased mortality rate of sepsis. Significant or intermittent endotoxemia may play an important role in this type of AKI, and sepsis is the most common cause of AKI in critically ill patients. There are two commonly used experimental models of Sepsis AKI [29]. One is to inject lipopolysaccharide (LPS), an endotoxin commonly used in experiments, into the abdominal cavity or blood to induce Sepsis [37]. The other is to induce AKI by replicating the clinical sepsis model by cecal ligation puncture (CLP) [38].

#### 2.2.3. Durgs/Toxins-AKI Models

Clinically used chemicals, such arsenic (As) aminoglycoside antibiotics (such as gentamicin (GM) [39]), antitumor drugs (such as cisplatin (CP) [40, 41] doxorubicin (DOX) [42] and methotrexate (MTX) [15]), As [43], folic acid [44] and glycerol [45, 46], are often used to induce AKI model due to their unavoidable side effects or obvious renal toxicity. Among them, glycerol causes rhabdomyolysis (RM). Clinically, RM is usually caused by hyperactivity, and AKI is the most common serious complication of RM. In addition, contrast-induced nephropathy induced by iodinated contrast media (CM) is also a type of acute kidney injury [47].

In these experimental models of AKI, neutrophil gelatinase-associated lipid carrier protein (NGAL) is significantly upregulated and heavily expressed in the kidney [48]. In this case, NGAL may inhibit nephrotoxicity by reducing apoptosis and increasing normal proliferation of renal tubular cells. Kidney injury molecule-1 (KIM-1) is a type of membrane penetrating protein that is significantly upregulated in the proximal tubules during AKI, and its extracellular domain is shed, which can be measured in urine [49]. As biomarkers of AKI damage, their levels are elevated in both urine and blood [50]. Similarly, renal functional biomarkers such as serum creatinine (Cr) and blood urea nitrogen (BUN), which are primarily excreted by glomerular filtration, are also upregulated as the glomerular filtration rate (GFR) decreases [51]. It is worth mentioning that these indicators are often used as clinical assistance in the diagnosis of AKI [49, 52].

Due to the complex pathogenesis of AKI, experimental models of AKI vary under different conditions. However, so far, some common pathogenesis has been found in various types of AKI, such as oxidative stress [53], excessive inflammation, mitochondrial damage [54], autophagy [55], and multiple forms of cell death [3] have been widely reported, but the search for new therapeutic targets and the discovery of new therapeutic agents to treat AKI is urgently needed.

## 3. Effects of Alkaloids from Chinese Herbs in AKI

A large number of studies have demonstrated that alkaloids extracted from natural Chinese herbs can reduce AKI biomarkers (such as Cr, BUN, KIM-1 and NGAL) [56]. More importantly, their renal protective effects may be related to antioxidant effect [57], improvement of mitochondrial damage [58], reduction of cell death [59], recovery of autophagy and reduction of inflammatory response [11], which has constructive significance for the discovery of drugs for the treatment of AKI from natural chemical components of Chinese herbal medicine. The pathways involved in these alkaloids have been summarized in Table 1.

### 3.1. Oxidative Stress (Nrf2/HO-1 Pathway)

Oxidative Stress refers to a state of imbalance between oxidation and antioxidants in vivo, which plays an important role in the development of AKI. Some renal stimulation leads to a decrease in the overall antioxidant capacity of enzymes in the body, including superoxide dismutase (SOD), catalase (CAT), glutathione (GSH), and glutathione peroxidase (GPX). This causes a series of reactive oxygen species (ROS) accumulation and oxidative stress. At the same time, ROS will react with biological macromolecules on the biofilm, resulting in lipid peroxidation products, such as malondialdehyde (MDA) and 4-hydroxynonenoic acid (4-HNE), will affect the normal physiological function and biological membrane [60–62]. This is also the main cause of kidney injury after acute nephrotoxicity.

*N*-acetylcysteine (NAC), an excellent source of sulfhydryl (SH), is characterized by an inhibition of oxidative stress. Because it can be converted into substances that stimulate GSH synthesis in AKI. Similar studies have found that NAC (500 mg/kg, i.p.), like Tetramethylpyrazine (TMP) (100 mg/kg, p.o.), reduces KIM-1 levels in rat models of AKI [43]. It has been reported that TMP can increase the level of GSH, SOD, and CAT induced by ROS production in renal cells induced by As or CP [43, 63, 64]. Oxidative stress is also the main molecular mechanism of I/R-AKI. Under I/R(or H/R) stimulation, BBR can significantly reduce MDA production, improve mitochondrial function, inhibit lipid peroxidation, and protect renal cells from the effects of oxidative stress [17, 65]. More strongly, BBR nanoparticles (2, 4 mg/kg, i.v.) have a similar effect and even superior renal protection and bioavailability [66]. A study also showed that intravenous injection of BBR(20, 40 mg/kg) can significantly reduce GM-induced lipid peroxidation of rat renal cells, upregulate SOD and GSH levels, and improve renal oxidative stress [67]. CHEN et al. further confirmed that BBR(5, 10, 20 mg/kg, i.v.) pretreatment could play a certain antioxidant role by reversing the changes in MDA, SOD, GSH and MDA levels caused by DOX, thus alleviating DOX induced liver and kidney damage [42].

In addition, in other experimental models of AKI, vinpocetine(Vin) [68], oxymatrine (OMT) [69], anisodamin [46], harmine [70], gelsemine [71] and leonurine [72] could up-regulate the levels of SOD, CAT, GSH and GPX, and reduce the contents of ROS and MDA, suggesting that these alkaloids could alleviate oxidative stress state and restore intracellular oxidative stress, thus playing a renal protective role.

Nuclear factor E2-related factor 2 (Nrf2) is a key nuclear factor in oxidative stress. Under basal conditions, Nrf2 inhibitor kelch-like ECH-associated protein 1 (Keap1) binds to it and preserves it in the cytoplasm. When oxidative stress occurs, Keap1 releases Nrf2, which translocates to the nucleus and binds to the antioxidant response element (ARE) element, an antioxidant response element, to activate the intracellular defense mechanism to counteract oxidative stress. The activation of Nrf2 induces downstream heme oxygenase-1 (HO-1) and NAD(P)H: quinone oxidoreductase 1 (NQO1), which can quickly neutralize, detoxify and remove oxidizing foreign bodies. The expression of HO-1 and NQO1 has both antioxidant and anti-inflammatory effects [46, 60, 73].

It was found that in Nrf2 knockout AKI mouse models, kidney damage was aggravated due to Nrf2 loss, which was mitigated by antioxidant treatment. Moreover, the application of Nrf2 activators in wild-type mice or rats significantly improved AKI caused by a variety of triggers. Thus, Nrf2 protects renal function by activating the antioxidant reduction system, thereby ameliorating tubular damage and tissue abnormalities [61].

When AKI occurs, the nuclear factor Nrf2 signaling pathway is inhibited, leading to the aggravation of oxidative stress and the destruction of the normal redox balance in cells. However, BBR [15], Vin [68] and OMT [69] can activate the Nrf2 defense pathway, which is manifested by high expression of Nrf2 and HO-1 in vivo and in vitro. Meanwhile, BBR can also reduce the expression of Keap1 gene. Similarly, TMP preconditioning can also upregulate the increase of Nrf2, HO-1, and NQO1 in the kidney of CP or As induced AKI rats. This explains the strong antioxidant capacity of these alkaloids by regulating the endogenous Nrf2/HO-1 system, while the decrease of Keap1 allows more Nrf2 to be free, thus providing more kidney protection [15, 74]. In vitro experiments, pre-treatment of NAC (10 mM) and TMP (50, 100 mM) also fully blocked the upregulation of HO-1 protein induced by As induced HK-2 cells, and inhibited ROS production [64]. In addition, TMP, other mechanisms involved in AKI, such as inflammation [63] and apoptosis [75], were also significantly improved.

### 3.2. Mitochondrial Damage

Mitochondrial dysfunction plays an important role in the pathogenesis of AKI. Mitochondria not only produce adenosine triphosphate (ATP) through oxidative phosphorylation, which provides the energy needed by cells, but also plays an important role in cell metabolism, cell apoptosis and other biological processes [76]. AKI, when it happens, damaged mitochondria increases more than mitochondrial autophagy mechanism to adjust the ability, or mitochondrial autophagy is suppressed, eventually lead to damaged mitochondria cannot keep clear of in time, accumulation within the cell, promote the formation of excessive ROS mitochondrial respiratory chain, promoting apoptosis factors increase, the cell oxidative stress, appear even rely on the apoptosis of mitochondria [77, 78].

GM demonstrated mitochondrial dysfunction in AKI by inhibiting the activity of NADH dehydrogenase (Complex-I) and cytochrome c (cyto c) oxidase (Complex-IV) of the mitochondrial respiratory chain. Restoring the changes of GM on the activity of Complex-I and Complex-IV, BBR maintained mitochondrial homeostasis to effectively delay the progression of kidney disease by maintaining mitochondrial homeostasis [17, 67].

### 3.3. Cell Death

Tubule repair and regeneration is considered the major event of renal recovery in AKI. Although the fatal damage is reversible, tubular cell death is accompanied by an inevitable loss of function of the affected cells.

Mechanically, cell death in AKI may be caused by well-described external and internal pathways and endoplasmic reticulum stress. The central meeting point of these pathways is the mitochondria, which become fragmented and sensitive to membrane permeability in the cellular stress response, leading to the release of cell death-inducing factors. Various forms of cell death are noteworthy: apoptosis in particular. In addition, other cell death pathways, such as ferroptosis, pyroptosis, and necroptosis, may also be involved in the pathophysiology of AKI.

#### 3.3.1. Ferroptosis

Ferroptosis is a new type of “regulatory cell death”, which is different from apoptosis, necrosis, and autophagy. Lipid peroxidation is considered to represent the downstream characteristics of ferroptosis, that is, the result of ferroptosis is the lack of an effective anti-peroxidation mechanism. The antioxidant system depends on the GSH system. GPX4 is the regulatory core enzyme of the GSH system, and its reduction will lead to ROS accumulation [79]. In addition, ferroptosis-suppressing protein 1 (FSP1), an oxidoreductase independent of the GSH system reduces coenzyme Q10, resulting in the production of lipophilic free radical trapping antioxidant (RTA), which prevents plasma membrane rupture after lipid peroxidation [80].

Ferroptosis is associated with the pathological process of many diseases, including AKI [81]. It has been found that conditional gene knockout of Gpx4 in the kidneys of mice can lead to AKI. In mice with AKI induced by many nephrotoxic drugs, ferroptosis inhibitor ferostatin-1 (FER-1) can reduce the levels of BUN and serum Cr, effectively restrain their oxidative stress and renal tubular cell death, thereby improving AKI [82–85]. Similar results were found in vitro [86].

Previous studies had reported that nuciferine from lotus leaf therapy weakened the increase of KIM-1 and NGAL, relieved renal fibrosis, and promoted renal regeneration in folic acid-induced AKI mouse model. These ameliorative effects of nuciferine may be related to its effect on cell ferroptosis, as nuciferine treatment evident reversed the expression levels of GPX4 and FSP1 mRNA and protein, while restoring iron metabolism. In vitro experiments, lactate dehydrogenase (LDH) content was indeed increased in HK-2 and HEK293T cells treated with RSL3 (a classical inducer of ferroptosis), and the cells showed distorted morphology. These phenomena were significantly reversed after the administration of lotus leaf line. The renal protective effect of nuciferine may be suggested by inhibiting ferroptosis [87].

#### 3.3.2. Apoptosis (Bax/Bcl-2/Caspase-3 Pathway)

**Figure 3:**
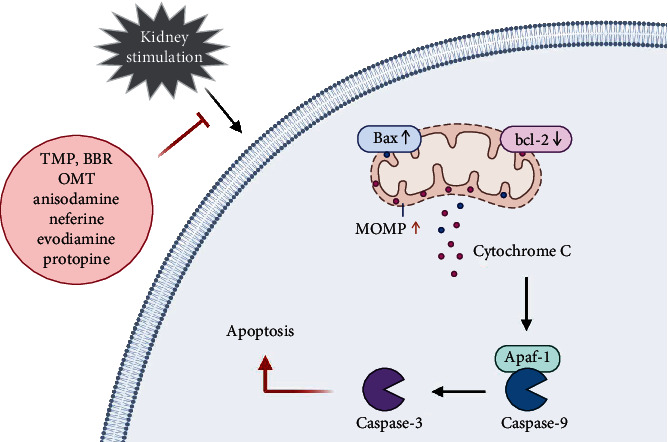
Some alkaloids inhibit apoptosis in AKI. Some alkaloids can improve AKI by affecting apoptosis in the mitochondrial pathway. (TMP, Tetramethylpyrazine; BBR, berberine; OMT, oxymatrine; MOMP, mitochondrial outer membrane permeability).

Apoptosis is mainly caused by the endogenous mitochondrial pathway among all kinds of renal toxin-induced AKI. In this pathway, cellular stress directly leads to increased mitochondrial outer membrane permeability (MOMP), which activates proapoptotic Bax protein and antiapoptotic Bcl-2 protein on mitochondria, thereby promoting the release of cyto c, which then binds with Apoptosis protease-activating factor-1 (Apaf-1) to activate caspase-9 and further activate caspase-3 to perform apoptosis (Figure 3).

Apoptosis is a common way of cell death in AKI, and it is also one of the channels through which alkaloids from Chinese herbal medicine play a role in renal protection. Many alkaloids, such as TMP [63, 75, 88, 89], BBR [15, 65, 66, 90], oxymatrine [69], anisodamine [46], neferine [87], evodiamine [91] and protopine [92], can improve AKI by affecting cell apoptosis. A number of studies have found that these alkaloids can protect renal function in a variety of AKI models by increasing the content of Bcl-2, reducing the expression of Bax, down-regulating the protein levels of caspase-9 and caspase-3 to inhibit apoptosis. Among these, both TMP and BBR inhibit the p38 mitogen-activated protein kinase (p38MAPK) signal transduction pathway that plays a key role in apoptosis [88, 89]。In addition, BBR can inhibit the transcription of p53-induced apoptosis gene activated by CP, reverse the phenomenon of cytoplasmic cyto c reduction and mitochondrial cyto c increase [65, 66], thereby blocking the Caspase-dependent apoptosis pathway [90].

#### 3.3.3. Pyroptosis

Cell pyrotosis is a new type of programmed cell death discovered and confirmed in recent years. It mainly relies on the activation of caspase-1/11 by cleavage gasdermin D (GSDMD), interleukin-1*β* (IL-1*β*), and interleukin-18 (IL-18) precursor. Activated GSDMD-N translocates to the membrane and forms holes, leading to continuous enlargement of the cell until the cell membrane ruptures. Large amounts of IL-1*β* and IL-18 are released to activate a strong inflammatory response [93].

Pyroptosis has been observed in many AKI models, such as those induced by I/R, CP, and LPS. It was found that LPS-induced renal NLRP3, caspase-1/pro-caspase-1, IL-1*β*, IL-1*β*, and GSDMD levels were increased in AKI mice, whereas Protopine decreased these protein levels [92]. It is suggested that one of the mechanisms of protopine on AKI may be through the pathway of cell pyroptosis.

#### 3.3.4. Necroptosis

Necroptosis is a novel cell death pathway which is different from apoptosis and necrosis and plays an important role in many disease models [94]. In glycerol-induced AKI, it was found that RIP3, which mediated necroptosis, was localized to the damaged proximal renal tubular epithelial cell peeling and necrotic cell membrane, but renal function and pathological changes were significantly improved after anisodamine treatment [80, 92]. Although the relationship between scopolamine and necroptosis in this study is not clear, it suggests that necroptosis is involved in renal cell death in AKI and may be an important target for the treatment of AKI.

### 3.4. Autophagy (PINK 1/Parkin Pathway)

Autophagy is a catabolic process in which autophagy lysosomes degrade most cytoplasmic contents. In this process, the autophagy marker light chain 3 (LC3) is synthesized, and LC3 I is lipidized to LC3 II through a ubiquitin-like system involving autophagy-related gene 7 (ATG 7) and autophagy-related gene 3 (ATG 3). LC3 II binds and is always localized to the autophagosome membrane in cells. It can reflect the amount of autophagy, and the ratio of LC3 II to LC3 I is a marker of autophagy [95].

Mitophagy is a selective autophagy process that specifically removes damaged or unwanted parts of mitochondria from cells. The most widely studied mechanism of mitophagy in renal tubule cells is mediated by the PINK 1/Parkin pathway [96, 97]. When mitochondrial damage occurs, PINK stabilizes and recruits Parkin to initiate autophagy. Mitochondrial membrane proteins lead to autophagy junction protein SQSTM1/p62 aggregation through the polyubiquitination of Parkin, which binds to LC3 via LC3- action region (LIR), thereby transferring the protein to the autophagy lysosomes for degradation. When autophagy occurs due to protein degradation, the level of p62 gradually decreases, so p62 is one of the markers protein for detecting autophagy activity [98, 99].

Studies on AKI have found that inhibitors of autophagy enhance the damage of renal tubular epithelial cells, whereas activators of autophagy show a protective effect, indicating that the removal of damaged mitochondria through mitophagy is an important mechanism for autophagy to provide renal protection [97, 100]. In CP-induced AKI mice and Renal epithelial cells (RTEC), BBR can increase LC3 II/LC3 I, PINK 1, and Parkin, and decrease the expression of P62 [90, 101]. These results indicate that BBR can mediate mitophagy in AKI through the activation of PINK1/Parkin signal and reduce the accumulation of damaged mitochondria in cells, which may be one of the pathways of alkaloids to alleviate AKI.

### 3.5. Inflammation

Inflammation compromise vascular and cellular mechanisms in response to tissue damage, so renal histopathological examination of AKI often shows increased neutrophilic infiltration. Manifested as proinflammatory factors, For example, the levels of tumor necrosis factor-*α* (TNF-*α*), interleukin-6 (IL-6), IL-1*β* are upregulated, and the levels of interleukin-10 (IL-10), the opposite anti-inflammatory factor, are decreased. These factors are expressed in both glomerular mesangial cells and renal tubule cells, and AKI shows an intense inflammatory response. The defense against inflammation is one of the most important anti-AKI mechanisms of alkaloids [102].

**Figure 4:**
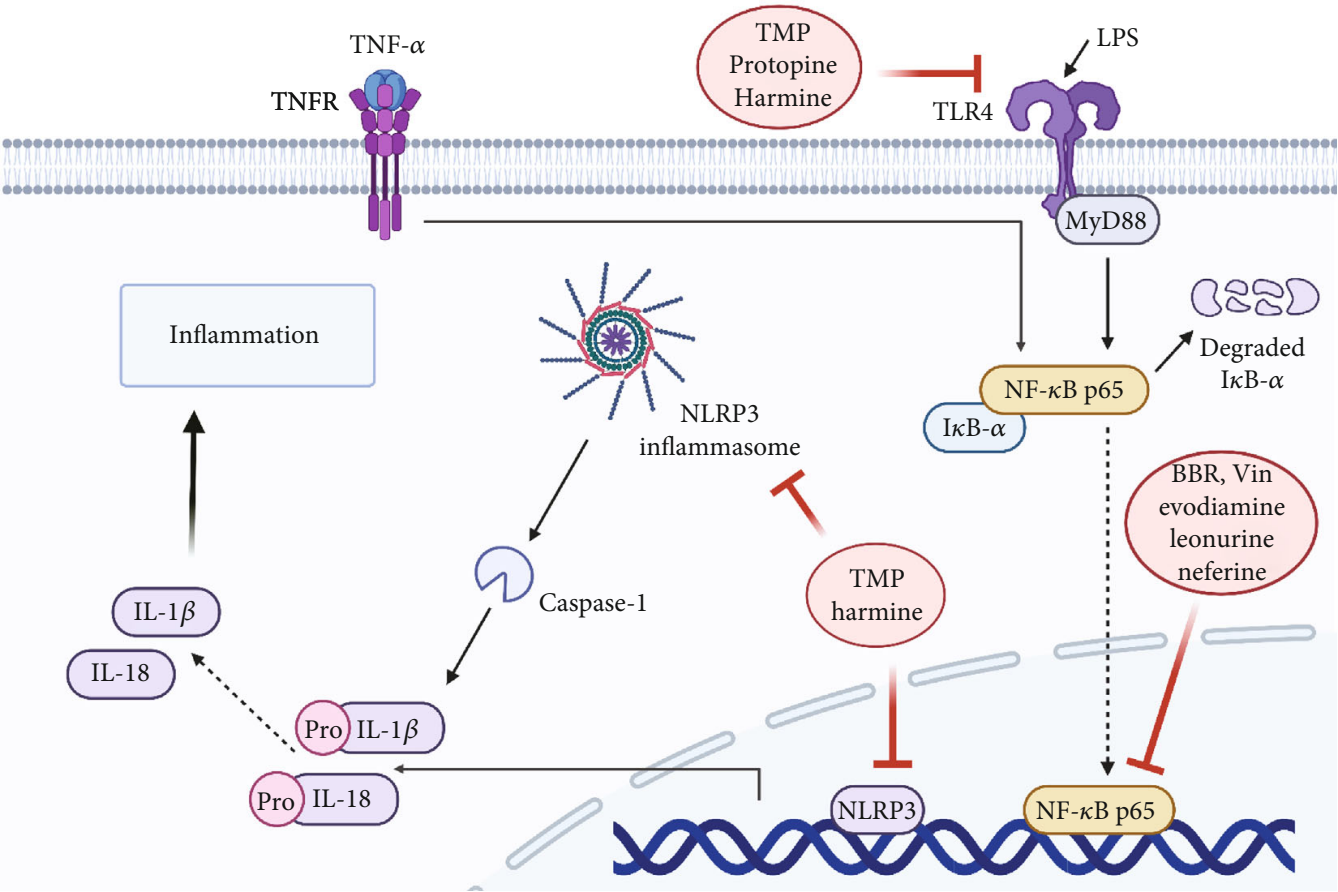
Some alkaloids inhibit cellular inflammation in AKI. The TLR4/NF-*κ*B and NLRP3 inflammasome pathways may be the primary mechanism by which some alkaloids inhibit inflammation in the treatment of AKI. (TMP, Tetramethylpyrazine; BBR, berberine; Vin, vinpocetine; OMT, oxymatrine).

Various alkaloids have been found to influence the changes in the levels of these inflammatory cytokines in different factor-induced AKI. For example, TMP [63], BBR [15, 90], Vin [68], protopine [92], evodiamine [91], harmine [70], neferine [103] and leonurine [72] can significantly reverse the massive release of TNF-*α*, IL-6, and IL-1*β* induced by AKI, among which proopioid protopine and evodiamine can also increase IL-10 level, regardless of LPS or I/R stimulation. Pre-treatment with anisodamine (1 mg/kg, i.p.) significantly reduced renal IL-6 protein levels in glycerol-treated rats. Anisodamine significantly reduced the mortality of glycerol-induced AKI rats and appeared to be more effective and less toxic [46], especially compared with atropine. The mechanisms involved are shown in Figure 4.

#### 3.5.1. TLR4/NF-*κ*B Signal Pathways

Nuclear factor kappa-B (NF-*κ*B) is an important mediator of the inflammatory response, which integrates extracellular stimulation with intracellular signal transduction pathways and plays a key role in the inflammatory events of AKI. NF-*κ*B is mainly activated by nuclear translocation, which consists of three sequential steps: phosphorylation/ubiquitination/acetylation, proteolytic degradation of inhibitor-*κ*B-*α* (I*κ*B-*α*), and nuclear translocation of NF-*κ*B. Most NF-*κ*B target genes are regulated by p65. The expression of various proinflammatory cytokines after NF-*κ*B activation can promote the NF-*κ*B pathway and eventually lead to renal insufficiency I [104].

Inhibition of NF-*κ*B activation may be an effective way to improve AKI inflammation. Treatment with BBR [15, 67, 90], leonurine [72], neferine [103], evodiamine [91] and Vin [68] significantly reduced the activation of NF-*κ*B and nuclear translocation of NF-*κ*B p65, and decreased phosphorylation and degradation of I*κ*B-*α*, further confirming their anti-inflammatory effects against GM and CP-induced nephrotoxicity.

Toll-like receptor 4 (TLR4), upstream of the NF-*κ*B pathway, is a classical LPS pathogen recognition receptor, which plays an important role in the innate immune system. TLR4 can activate myeloid differentiation factor 88 (MyD88) and NF-*κ*B. In the AKI models, the level of TLR4 in renal tubular epithelial cells were significantly increased because of activation dependent on HSP70. As the main ligand of TLR4, HSP70 plays an indispensable role in immune response and inflammatory response. It can activate TLR4-mediated signaling pathway and promote the production of proinflammatory cytokines and chemokines [105].

TMP [63], protopine [92] and harmine [70] inhibit the expression of TLR4 and MyD88. Especially, TLR4/NF-*κ*B signaling pathway is affected. The inhibition of TLR4 may be related to the regulation of HSP70, but relevant experimental proof is still needed. In addition, peroxisome proliferator-activated receptor -*γ* (PPAR-*γ*) agonists have been described, and TMP also promotes the expression and protein levels of the powerful TLR4 inhibitor PPAR-*γ* gene [63, 70, 92]. In summary, these alkaloids have a protective effect against acute kidney injury by reducing the inflammatory response. Inhibition of TLR4/NF-*κ*B pathway and inhibition of the NACHT, LRR, and PYD domain-containing protein 3 (NLRP3) inflammasome pathways may be one of the mechanisms for the treatment of AKI.

#### 3.5.2. NLRP3 Inflammasome Pathway

The nucleotide-binding oligomerization domain receptors (NOD-like receptors, NLR) protein family is a pluralistic family of cytoplasmic innate immune receptors. The role of NLR protein in the inflammatory response is mainly the formation of NLRP3 inflammasome and NOD2, which can activate the inflammatory caspase through the transcription of NF-*κ*B to activate the proinflammatory cytokines [106]. NOD2 and NLRP3 are widely involved in the development of renal disease [107].

NLRP3 is an inflammasome that activates caspase-1 and thus activates IL-1*β* and IL-18 to form NLR. NLRP3 inflammasomes assemble in response to various stimuli, including during the development of AKI. Similarly, hypoxia-inducible factor-1*α* plays a major role in the body's inflammatory response to AKI. NLRP3 inflammasome activity is maintained by the hypoxia-inducible factor-1*α* (HIF-1*α*) pathway. It has been found that TMP may reduce the expression of NLRP3 by decreasing the expression of HIF-1*α* [75]. In addition, harmine can inhibit the expression of NLRP3, caspase-1 and IL-1*β* in LPS-AKI [70].

As a well-defined member of the NLR family, NOD2 is responsible for the high expression of inflammatory factors, which is mainly dependent on the activation of NF-*κ*B. This is also evidence that TMP protects renal injury after AKI by inhibiting NOD2-mediated inflammation [108].

#### 3.5.3. Klotho Protein

Klotho is a one-way transmembrane protein with two forms of transmembrane and soluble Klotho (SKL), which is mainly synthesized in distal renal tubules. SKL may protect the kidneys by being anti-inflammatory. Klotho expression decreased after multiple factors induced AKI. More importantly, TNF-*α* downregulates Klotho expression in renal tubule cells through an NF-*κ*B-dependent mechanism. In I/R and LPS-induced AKI models, neptoline may inhibit the NF-*κ*B pathway by increasing the expression of Klotho. In combination with those previously described, it is suggested that neptoline directly and indirectly affects the activation of NF-*κ*B and exerts anti-inflammatory effects in AKI [103]。

## 4. Nephrotoxicity of some Alkaloids

Recent studies have found that there are some nephrotoxic alkaloids from natural Chinese herbs, in addition to those with nephroprotective effects [109, 110]. Examples include Strychnine or Brucine, separated from *Strychnos nux-vomica* Linn, and Aconitine, the main toxic substance in Aconitum *Carmichaeli* Debx [111]. There are also major alkaloids extracted from *Tripterygium Regelii* Sprague Et Takeda [112], whose renal toxicity has been mentioned in previous reviews [113]. It is well known that the toxicity of aconitum is a famous Chinese herbal medicine, which need to pass special processing to alleviate the toxicity of medicinal [114]. A key part of the toxicity of aconitine, a diester alkaloid, is the ester bond in the molecule. Aconitine (1.46 mg/kg) can inhibit myocardium and lead to renal ischemia and hypoxia, which can cause degeneration and apoptosis of some renal tubular epithelial cells [115]. Similarly, toxic doses of strychnine also severely damage the kidneys. In vitro, Strychnine inhibited the proliferation of human proximal renal tubular epithelial cells (20-40 *μ*g/ml), and showed significant toxicity to HK-2 cells (80-160 mg/ml) [113]. In animal toxicity tests, it was found that long-term administration of tripterygium alkaloids (9.97 mg/g) for four weeks could cause damage and even necrosis of renal tubular epithelial cells in SD male rats [116].

In addition, the inhibition of organic anion transporters by alkaloids may be one of the mechanisms of nephrotoxicity. The main function of organic anion transporters(OATs) is to transport organic anions in the blood to the epithelial cells of proximal renal tubules. OAT1, OAT3, and OAT4 have been confirmed to be expressed mainly in the proximal convoluted tubules of the kidney, and they mediate the excretion of a series of small molecular hydrophilic organic anion compounds through the kidney [117]. And when the failure of renal function, its transmission function will be abnormal, thus affecting drug excretion [118]. It was found that strychnine and aconitum alkaloids inhibited OTA1 and OTA3 functions and caused kidney damage [119].

On the one hand, the effect of nephrotoxic alkaloids on renal function is very dose-dependent, even though some alkaloids have a protective effect on AKI. Once the dose is too high, conversely, it can also be highly toxic to the kidneys. In addition, they inhibited OATs on the proximal curved tubules of the kidney, which was similar to one of the roles of CP in the induction of AKI. However, due to the numerous structural classifications of alkaloids and lack of commonality, it is difficult to explain the positive or negative effects of alkaloids on kidney from the structural types. Therefore, the structure-activity relationship of alkaloids in the treatment of AKI is not summarized in this review.

## 5. Discussion

The structure of a compound is directly related to biological activity, so the study and modification of the structure plays a pivotal role to find the key active groups. The pathogenesis of AKI is extensive, therefore, further study on the structure-activity relationship of various natural alkaloids will help to find a new drug for AKI.

Pathologically, AKI is characterized by renal tubular cell injury or death, inflammation, and vascular dysfunction [120].

**Figure 5:**
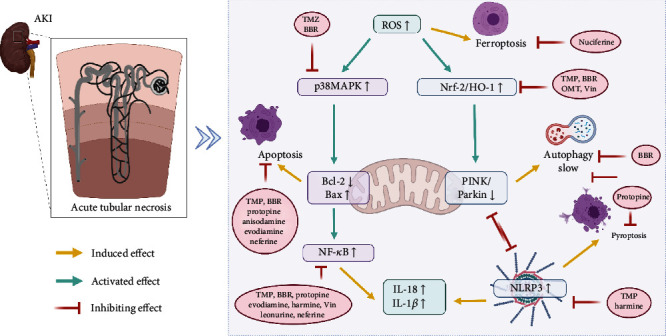
Different targets of alkaloids in AKI. AKI, acute kidney injury; TMP, Tetramethylpyrazine; BBR, berberine; Vin, vinpocetine.

When the body is subjected to a certain level of stimulation, for example, the high production of ROS causes oxidative stress, which activates the MAPK family to promote inflammatory responses and cell death. In many studies, alkaloids have been found to activate the Nrf2/HO-1 antioxidant pathway when AKI occurs, reducing cellular ferroptosis and speeding up clearance by enhancing mitochondrial autophagy. Also, mitochondrial autophagy can affect the production of NLRP3 inflammatory vesicles, thereby decreasing inflammation and pyroptosis. In addition, the antioxidant effect of alkaloids also inhibited the activation of MAPK family, exerting anti-inflammatory and reducing apoptosis. Therefore, active substances with strong antioxidant effects in AKI may affect other mechanisms causing renal injury based on the cellular level, thus exerting more advantageous renal protection. Current studies have shown that different alkaloids have different targets for the improvement of AKI, as shown in Figure 5.

Alkaloids from Chinese herbs have a variety of activities, improving AKI plays only a small role in them, and there are more pharmacological effects and action targets to be explored. Furthermore, all results are based on cell/animal experiments and have no clinical application. Therefore, the mechanism of action of alkaloids in AKI needs to be further investigated.

Many of the above studies have a number of particularly noteworthy linkages that tie together the main mechanisms by which alkaloids improve AKI in a relatively complete small but progressive cycle. The pathways regulated by various targets in the human body are extremely complex. Researchers have endeavored to sort out as clearly as possible the mechanisms identified in the pathogenesis of AKI, thus gaining insight into the causes behind AKI will provide a better pathway for the development of drugs to treat AKI in the clinic.

## Figures and Tables

**Table 1:** Pathways involved in the amelioration of AKI by alkaloids.

Classification	Name	Source	Dose/ Concentration	Mechanisms	Models (in vivo/vitro)	Clinical etiology (mimic)
Pyrazine alkaloids	**Tetramethylpy-razine** **(TMP)/Ligustrazine**	*Ligusticum wallichii Franchat*	100 mg/kg, i.p.	Oxidative stress	Rats, GM ***(in vivo)*** [64]	Drugs/toxics
			50, 100 *μ*M	Oxidative stress (Nrf2/HO-1)	HK-2 cells, as *(in vitro)* [43]	Drugs/toxics
			50, 100 mg/kg, i.p.	Oxidative stress (Nrf2/HO-1), inflammation (TLR4/NF-*κ*B), apoptosis	Rats, CP *(in vivo)* [63]	Drugs/toxics
			40 mg/kg, i.p. *(in vivo)* 30, 50 *μ*M *(in vitro)*	Inflammation (NOD2), apoptosis, autophagy	Rats, I/R *(in vivo)*, NRK-52E cells, H/R *(in vitro)* [108]	Surgery
			40 mg/kg, i.p.	Inflammation (NLRP3), apoptosis (caspase-3)	Rats, I/R *(in vivo)* [75]	Surgery
			30, 60 mg/kg, i.v.	Apoptosis (caspase-3)	Mice, CLP *(in vivo)* [89]	Sepsis
			80 mg/kg, i.p.	Apoptosis (Bax/bcl-2)	Rats, CM *(in vivo)* [88]	Drugs/toxics

Isoquinoline alkaloids	**Berberine** **(BBR)**	*Berberis vulgaris*	10 *μ*M	Oxidative stress, mitochondrial damage, apoptosis (Bax/bcl-2/caspase-3)	HK-2 cells, H/R *(in vitro)* [65]	Surgery
			1, 2, 3 mg/kg, i.g.	Oxidative stress (Nrf2/HO-1), apoptosis (caspase-3), inflammation (NF-*κ*B), autophagy (LC3B)	Mice, CP *(in vivo)* [90]	Drugs/toxics
			20, and 40 mg/kg; p.o.	Oxidative stress, apoptosis, inflammation, mitochondrial damage	Rats, GM *(in vivo)* [67]	Drugs/toxics
			20, 40 mg/kg, p.o.	Oxidative stress, apoptosis (Bax/bcl-2/caspase-3), inflammation	Rats, I/R *(in vivo)* [17]	Surgery
			Nanoparticles, 2, 4 mg/kg, i.v.	Oxidative stress, apoptosis (Bax/bcl-2/caspase-3),	Rats, I/R *(in vivo)* [66]	Surgery
			5, 10, 20 mg/kg, i.v.	Oxidative stress	Rats, DOX *(in vivo)* [42]	Drugs/toxics
			50 mg/kg, p.o.	Oxidative stress (Nrf2/Keap1), apoptosis(Bax/bcl-2/caspase-3), inflammation (NF-*κ*B)	Rats, MTX *(in vivo)* [15]	Drugs/toxics
			5, 10 mg/kg, i.p. *(in vivo)* 1, 2, 4 *μ*M *(in vitro)*	Autophagy (PINK 1/Parkin)	Mice, CP *(in vivo)*, NRK-52E cells, CP *(in vitro)* [101]	Drugs/toxics

	**Protopine**	*C. Humosa Migo*	5 mg/kg, i.g.	Apoptosis (Bax/bcl-2/caspase-9/caspase-3), inflammation (TLR4/NF-*κ*B), pyroptosis	LPS, mice *(in vivo*) [92]	Sepsis
	**Neferine**	*Nelumbo nucifera*	20 mg/kg, i.p. *(in vivo)* 2, 4, 8 *μ*M *(in vitro)*	Inflammation(NF-*Κ*B, klotho), apoptosis (Bax/bcl-2/caspase-3)	Mice, I/R *(in vivo)*, NRK-52E cells, H/R or LPS *(in vitro)* [103]	Surgery, sepsis
Indole alkaloids	**Vinpocetine** **(vin)**	*Catharanthus roseus*	5 mg/kg, i.p. *(in vivo)* 3 *μ*/ml *(in vitro)*	Oxidative stress (Nrf2), inflammation(NF-*κ*B)	Mice, CP *(in vivo)*, HK-2 cells, CP *(in vitro)* [68]	Drugs/toxics
	**Gelsemine**	*Gelsemium elegans*	5, 25 mg/kg, p.o.	Oxidative stress	Rats, CP*(in vivo)* [64, 71]	Drugs/toxics
	**Evodiamine**	*Euodia rutaecarpa (Juss.)Benth*	10 mg/kg, i.p.	Oxidative stress, inflammation (NF-*κ*B), apoptosis (caspase-3)	Rats, I/R [91]	Surgery

Pyridine alkaloids	**Oxymatrine (OMT)**	*Sophora flavescens Ait,*	150 mg/kg, i.p.	Oxidative stress(Nrf2/HO-1)	Rats, I/R [69]	Surgery

Tropane alkaloids	**Anisodamine**	*Scopolia tangutica* *Maxim*	1 mg/kg, i.p.	Oxidative stress, inflammation, apoptosis(caspase-3)	Rats, glycerol [46]	Drugs/toxics

*β*-Carbine alkaloids	**Harmine**	*Peganum harmala L.*	25, 50 mg/kg, i.g.	Inflammation (TLR4/NF-*κ*B, NLRP3)	Mice, LPS [70]	Sepsis

Organic amine alkaloids	**Leonurine**	*Leonurus cardiaca*	50 mg/kg, p.o.	Oxidative stress, inflammation (NF-*κ*B)	Mice, LPS [72]	Sepsis

Aporphine alkaloids	**Nuciferine**	*Nelumbo nucifera*	30 mg/kg, i.g.	Ferroptosis	Mice, folic acid [87]	Drugs/toxics

## Abbreviations

AKI: Acute kidney injury
ICU: Intensive care unit
BBR: Berberine
ATN: Acute tubular necrosis
AIN: Acute interstitial glomerulonephritis
I/R: Ischemia/reperfusion
H/R: Hypoxia-oxygen-rich
LPS: Lipopolysaccharide
CLP: Cecal ligation puncture
GM: Gentamicin
CP: Cisplatin
DOX: Doxorubicin
MTX: Methotrexate
As: Arsenic
RM: Rhabdomyolysis
CM: Contrast media
NGAL: Neutrophil gelatinase-associated lipid carrier protein
KIM-1: Kidney injury molecule-1
Cr: Creatinine
BUN: Blood urea nitrogen
GFR: Glomerular filtration rate
SOD: Superoxide dismutase
CAT: Catalase
GSH: Glutathione
GPX: Glutathione peroxidase
ROS: Reactive oxygen species
MDA: Malondialdehyde
4-HNE: 4-hydroxynonenoic acid
TMP: Tetramethylpyrazine
Vin: Vinpocetine
OMT: Oxymatrine
Nrf2: Nuclear factor E2-related factor 2
Keap1: Kelch-like ECH-associated protein 1
ARE: Antioxidant response element
HO-1: Heme oxygenase-1
NQO1: NAD(P)H: Quinone oxidoreductase 1
ATP: Adenosine triphosphate
cyto c: Cytochrome c
FSP1: Ferroptosis-suppressing protein 1
RTA: Radical trapping antioxidants
FER-1: Ferostatin -1
LDH: Lactate dehydrogenase
MOMP: Mitochondrial outer membrane permeability
Apaf-1: Apoptosis protease-activating factor-1
p38MAPK: p38 mitogen-activated protein kinase.
GSDMD: gasdermin D.
IL-1*β*: Interleukin-1*β*
IL-18: Interleukin-18
LC3: Light chain 3
ATG 7: Autophagy-related genes 7
ATG 3: Autophagy-related genes 3
LIR: LC3- action region
RTEC: Renal epithelial cells
TNF-*α*: Tumor necrosis factor-*α*
IL-6: Interleukin-6
NF-*κ*B: Nuclear factor kappa-B
I*κ*B-*α*: Inhibitor-*κ*B-*α*
TLR4: Toll-like receptor 4
MyD88: Myeloid differentiation factor 88
PPAR-*γ*: Peroxisome proliferator-activated receptor -*γ*
NLRP3: The NACHT, LRR, and PYD domains-containing protein 3
NLR: NOD-like receptor, the nucleotide-binding oligomerization domain receptors
HIF-1*α*: Hypoxia-inducible factor-1*α*
SKL: Soluble klotho
NAC: N-acetylcysteine
SH: Sulfhydryl
OATs: Organic anion transporters.

## Supplementary Materials

Supplementary Materials Graphical abstract (TMP, Tetramethylpyrazine; BBR, berberine; AKI, acute kidney injury).
