# Supplementary figures and images for: Several Alkaloids in Chinese Herbal Medicine Exert Protection in Acute Kidney Injury: Focus on Mechanism and Target Analysis
Source: Oxid Med Cell Longev. 2022 May 13;2022:2427802. doi: 10.1155/2022/2427802 (PMC9122709; doi:10.1155/2022/2427802)

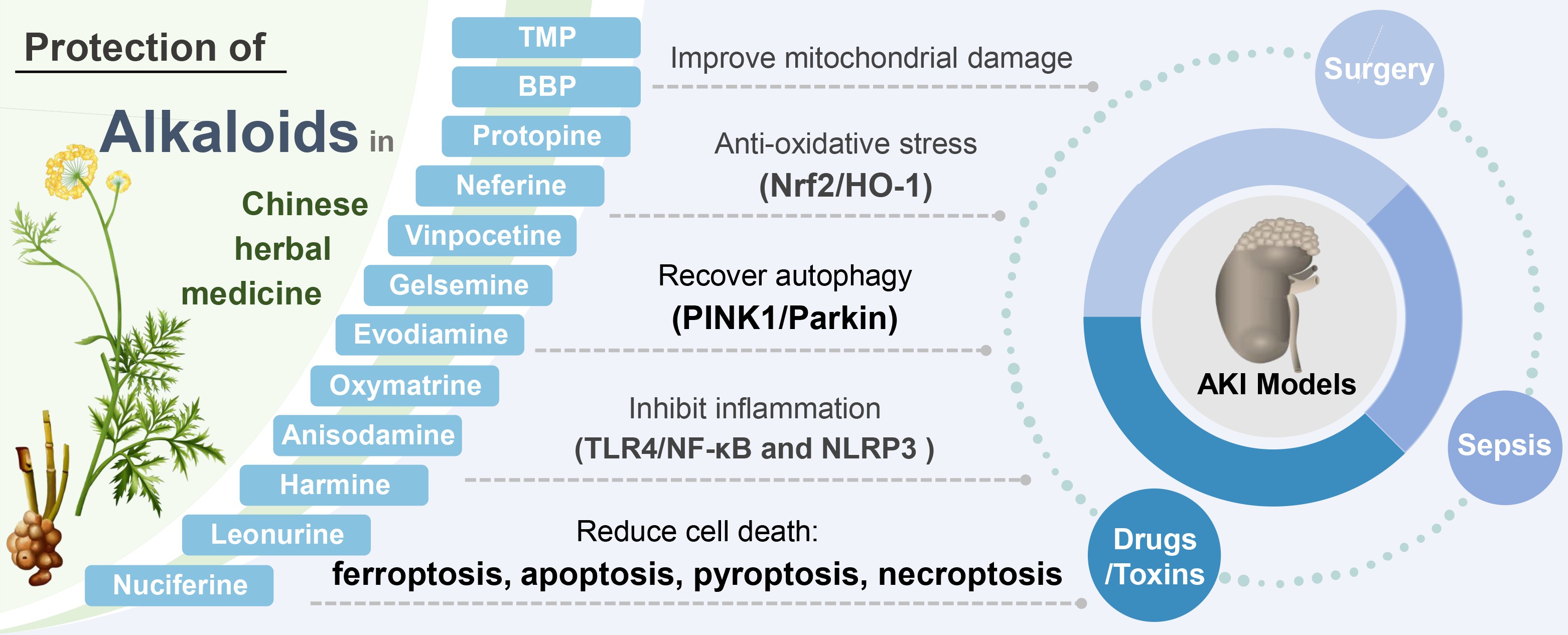

Supplement: Supplementary Materials — Graphical abstract (TMP, Tetramethylpyrazine; BBR, berberine; AKI, acute kidney injury). [file 2427802.f1.jpg]
